# Bridging, Braiding, and Weaving Indigenous and Western Science to Understand and Make Predictions About Weather and Climate Change

**DOI:** 10.1002/ece3.72085

**Published:** 2025-09-24

**Authors:** Keith Chaulk, Myrle Ballard, Stewart Hill, David Wolfrey, Mina Campbell, Mike Sutherland, Solomon Wawatie, Len Auger

**Affiliations:** ^1^ Indigenous Science Division Environment and Climate Change Canada Greater Napanee Ontario Canada; ^2^ Department of Earth, Energy, and Environment University of Calgary Calgary Alberta Canada; ^3^ Tier II Indigenous Science and Sustainability Science Calgary Alberta Canada; ^4^ Manitoba Keewatinowi Okimakanak, Inc Winnipeg Manitoba Canada; ^5^ Retired Rigolet Newfoundland and Labrador Canada; ^6^ Labrador Interpretation Centre Government of Newfoundland, and Labrador North West River Newfoundland and Labrador Canada; ^7^ Peguis Consultation & Special Projects Inc Peguis Manitoba Canada; ^8^ Retired Maniwaki Quebec Canada; ^9^ Auger Safety Consulting and Training Grand Prairie Alberta Canada

**Keywords:** Bridging, Braiding, and Weaving, climate change, Indigenous Science, Knowledge Holders, weather prediction

## Abstract

In this study, Indigenous Knowledge Holders were gathered in a collaborative forum to discuss weather, in particular, Indigenous Science knowledge related to severe weather, climate change, and weather prediction methods. Weather forecasting methods that arose in discussion were further explored based on a framework of *Bridging*, *Braiding*, and *Weaving* knowledge systems. Numerous Indigenous Science‐based weather prediction methods were documented, with most lending themselves to follow‐up testing. The Knowledge Holders emphasized that Indigenous Science is an important component within the broader domain of Indigenous Traditional Knowledge, with current and historic empirical and holistic insights into natural phenomena. There was consensus among the Knowledge Holders that Indigenous Science should not be perceived as subordinate to Western methodologies but instead viewed as a complementary and equivalent knowledge system. This paper underscores the importance of a co‐produced approach to research where Indigenous Knowledge Holders share their expertise to enrich and inform Western research design, including the interpretation of study results and the implementation of subsequent policy and decision‐making. We provide examples of *Bridging*, *Braiding*, and *Weaving* Indigenous Science using weather‐related phenomena, such as animal behavior, atmospheric patterns, and plant growth indicators, while also offering insights into the impacts of climate change and severe weather.

## Graphical abstract

This paper examines Indigenous weather science through a workshop with Indigenous Knowledge Holders, documenting traditional weather prediction methods and their responses to climate change and severe weather. Using the bridging, braiding, and weaving framework, it highlights the complementary nature of Indigenous and Western science. The study emphasizes the importance of co‐producing research with Indigenous experts to strengthen environmental research and decision‐making.

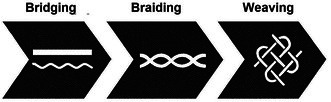

## 1 Introduction

Indigenous knowledge systems have long offered valuable insights into understanding environmental patterns, including weather phenomena (Chand et al. 2014; Egeru 2012; Orlove and Tosteson 1999). Indigenous knowledge systems are often collectively labeled as Traditional Knowledge and/or Indigenous Knowledge and have been used by Indigenous peoples globally for thousands of years to aid survival and enrich society (Reyes‐García et al. 2021; Petzold et al. 2020).

Within the framework of Indigenous Traditional Knowledge, there is an increasing recognition of Indigenous Science (Kimmerer 2021; Kolopenuk 2020; Michell et al. 2008). Cajete (2000) describes Indigenous Science as a comprehensive and holistic approach to understanding the natural world that has evolved over millennia through close observation and intimate knowledge of local environments. Meanwhile, Snively and Corsiglia (2000) describe Indigenous Science as encompassing a broad spectrum of knowledge, including plant, animal, and weather insights, and as intertwined with cultural, spiritual, and ecological aspects of Indigenous life.

The Indigenous‐led Indigenous Science Division (ISD) of Environment and Climate Change Canada (ECCC) currently defines Indigenous Science as a distinct, contemporary but time‐tested, empirical, and methodological knowledge system (ISD 2024). More specifically, ISD describes Indigenous Science (IS) as a culturally specific method of accumulating knowledge, refining hypotheses, and changing practices by drawing from Indigenous (First Nations, Inuit, and Métis) Peoples' deep understanding of the natural world. Indigenous Science can be viewed as a subset of Traditional Knowledge, with a specific focus on the physical world (air, water, plants, animals, weather, etc.). Indigenous Science emphasizes repeatable and empirical observations that are both contemporary and rooted in a long history of interaction with the physical environment. Indigenous Science is adaptable based on new information and changing conditions and is testable through practice, but it is interpreted through a holistic lens.

That said, Indigenous Knowledge, Traditional Knowledge, and Indigenous Science are often used interchangeably. In this paper, it may help to view Traditional Knowledge as the larger knowledge system in which Indigenous Science is situated. Although there is a large and growing body of information within the academic literature dealing with Indigenous Traditional Knowledge, there has been much less published on the topic of Indigenous Science. For example, Nadasdy (1999) indicated that Indigenous voices and expert opinions were historically given significantly less weight when compared to input that originated and more closely aligned with a Western European scientific paradigm. Similarly, Dickison (2009) reported similar condescending views and negative attitudes toward Indigenous Knowledge. Widespread views such as these are unfortunate because Indigenous Science can play a pivotal role in government policymaking and program implementation in key areas such as natural resource management, climate change adaptation, emergency preparedness, and research policy.

Regardless of terminology, Indigenous perspectives in the development and implementation of programs and policies that directly impact Indigenous communities have historically been sidelined (Royal Commission on Aboriginal Peoples 1996; Alfred 2005; Truth and Reconciliation Report 2015). Recently, Indigenous peoples in Canada have been achieving a greater voice in decisions that impact their communities (Coulthard 2007; Procter and Chaulk 2013; Dafnos et al. 2016; Inuit Tapiriit Kanatami 2018; Pfeifer 2018). For example, there is a growing body of legal decisions that deal with Indigenous rights, interests, and titles (R. v. Sparrow 1990; Delgamuukw v. British Columbia 1997; Tsilhqot'in Nation v. British Columbia 2014), along with the settlement of comprehensive land claims (i.e., Nunavut Land Claims Agreement 1993; Labrador Inuit Land Claim Agreement 2005; Tsawwassen First Nation Final Agreement Act 2008), the Truth and Reconciliation Report (2015), and prior to that, the Royal Commission on Aboriginal Peoples (1996). In addition, in 2021, the Government of Canada gave Royal Assent to the United Nations Declaration on the Rights of Indigenous Peoples (United Nation 2007; Canada 2021). Each of these, to various degrees, promotes Indigenous self‐governance, self‐determination, and/or new power structures within national and sub‐national jurisdictions that often require the inclusion of Indigenous Knowledge in decision‐making. Nonetheless, there is still much work to be done, as decision‐makers struggle with new and emerging requirements to incorporate Indigenous views and perspectives into their Western Science‐based practices (Nadasdy 1999; Parter and Skinner 2020). The ISD within the Science and Technology Branch of ECCC is working to assist this progress with a mandate to develop and apply an Indigenous lens to ECCC's science, policy, and program activities to better inform and enhance decision making. In this paper, we try to focus on Indigenous Science using the three‐tiered approach of *Bridging, Braiding, and Weaving* (Snively and Williams 2008; Snively and Wanosts'a7 L 2016; Hernandez and Spencer 2020; Indigenous Science Division 2024).

**FIGURE 1:**
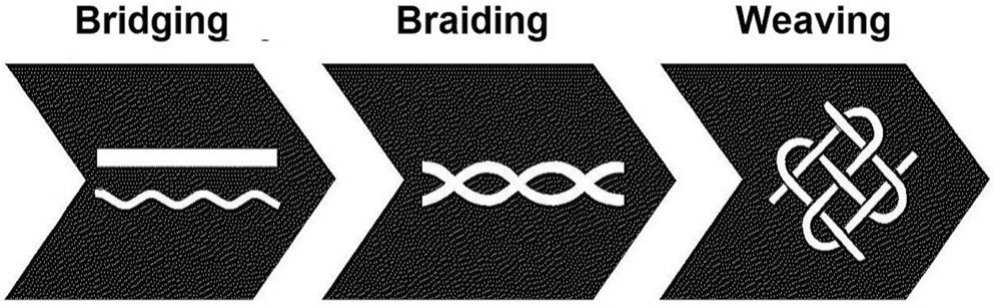
Depicting the *Bridging*, *Braiding*, and *Weaving* framework (Indigenous Science Division 2024). Each symbol represents a framework step. While the steps can overlap and interact, typically each step builds off the one prior, with the process becoming more complex as it progresses. *Bridging* is described as processes and activities that bring together Indigenous and Western Science to convey mutual awareness and respect. *Braiding* is the intertwining of knowledge systems for a more comprehensive understanding. *Weaving* is the application of Indigenous methodologies and paradigms alongside Western approaches to research. *Weaving* is broader in scope than *Bridging* or *Braiding* and requires the involvement of Indigenous Peoples and their knowledge in the study design, resourcing, program implementation, data collection, data interpretation, reporting, and, if applicable, any subsequent policy development, decision‐making, and governance arising from the research activity. The *Bridging*, *Braiding*, and *Weaving* framework was initially developed by Dr. Myrle Ballard in 2022, during her tenure as the inaugural Director of the Indigenous Science Division of ECCC.

The ISD of ECCC (2024) describes *Bridging*, *Braiding*, and *Weaving* as a three‐step framework, with each step being more complex but building off any prior steps (Figure 1). In some respects, the framework of *Bridging*, *Braiding*, and *Weaving* is closely related to the concept of Two‐Eyed Seeing (Bartlett et al. 2012). *Bridging* can be described as processes and activities that bring together Indigenous and Western Science to convey mutual awareness and respect. *Bridging* is about fostering awareness, understanding, and recognition of Indigenous Science as distinct from and complementary to Western scientific approaches. *Bridging* can be accomplished through a combination of engagement activities and ongoing relationship building (i.e., through repatriation of lands, materials, knowledge and development and sharing of educational resources). The ISD of ECCC describes *Braiding* as the intertwining of knowledge systems for a more comprehensive understanding. *Weaving* is the application of Indigenous methodologies and paradigms alongside Western approaches to research, including any subsequent applications to policy development and decision‐making (ISD 2024).

Each of these three framework steps can be in a range of states, from absent to partial to fully realized. For example, within the context of *Bridging*, it is possible to be partially realized if someone is made aware of a phenomenon recognized by Indigenous Science, but that bridge is only partially realized if those observations and insights are not respected or are dismissed outright. In this case, awareness has been achieved, but respect is absent. This does not mean that skepticism cannot be present in the context of *Bridging*, but the recipient of the information should be open‐minded to receive supporting evidence, including the form of the evidence. This paper aims to describe recently collected Indigenous Science related to weather, and the authors then apply the framework of *Bridging*, *Braiding*, and *Weaving* to demonstrate synergies and how Indigenous Science can be applied in conjunction with Western Science.

## 2 Methods

### 2.1 Workshop Rationale

To better understand Indigenous Science in practice, ISD pinpointed Indigenous Science as it relates to weather as a critical theme that overlapped with ECCC's responsibilities in meteorology and climate change surveillance. Workshop planning began with a literature review to better understand the state of written work regarding Indigenous Science in Canada as it related to weather. While a few papers related to Indigenous Knowledge and weather were noted (Riedlinger and Berkes 2001; Gearheard et al. 2010; Weatherhead et al. 2010; Alexander et al. 2011), overall, the review uncovered a notable gap in documentation of weather‐specific knowledge, irrespective of its classification as Indigenous Knowledge, Traditional Knowledge, or Indigenous Science.

Confronted with the scarcity of documented information, ISD set in motion the organization of an Indigenous Science scoping workshop on the topic of weather. This concerted effort materialized in Ottawa on October 25–26, 2023, assembling six Indigenous Knowledge Holders (in this paper sometimes referred to as Knowledge Holders) from different regions of Canada and from different Indigenous backgrounds (First Nations and Inuit, see Table 1). Knowledge Holders were identified through regional networks of ISD. Initially, the workshop planned to include approximately 26 Indigenous Knowledge Holders (i.e., two from each Province and Territory), but the funding was reduced after the initial set of invites were sent out and accepted, and the workshop proceeded with those Knowledge Holders who had agreed to participate. From the beginning, the workshop's methodology was co‐developed with the Knowledge Holders and was designed to increase awareness and respect for Indigenous Science (*Bridging*), while seeking opportunities for further *Braiding* and *Weaving* Indigenous and Western Science.

**TABLE 1:** Summary of workshop participants, their backgrounds, and roles (if any) in the workshop and authorship of current paper.

Name, role and background
*Solomon Wawatie*: *Anishinaabe Elder and Knowledge Holder* of the Bear Clan and lives in Quebec, Canada. He was also *Workshop Elder*, and *Co‐Author* and he provided input into workshop agenda and thematic areas, led the opening and closing of the workshop, active participant in all aspects of the workshop discussions, involved in writing and reviewing paper
*Mina Campbell*: Labrador Inuit *Knowledge Holder* and beneficiary of the Labrador Inuit land claim. She is senior curator of the Labrador Interpretation Center. She was *Co‐Author* and provided input into workshop agenda and thematic areas. Active participant in all aspects of the workshop discussions, involved in writing and reviewing paper
*Stewart Hill*, PhD: *Cree Scholar and Knowledge Holder* from God's Lake First Nation, Manitoba, Canada. His PhD research focused on Indigenous water and land governance. He was a *Co‐Author* and provided input into workshop agenda and thematic areas. Active participant in all aspects of the workshop discussions, involved in writing and reviewing paper
*Mike Sutherland*: He is a member of the *Peguis First Nation in Manitoba, Canada*. He is a well‐respected *Knowledge Holder* and is also Director of the Peguis Consultation & Special Projects Unit. He was a *Co‐Author* and provided input into workshop agenda and thematic areas. Active participant in all aspects of the workshop discussions, involved in writing and reviewing paper
*David Wolfrey: Labrador Inuit Knowledge Holder* and beneficiary of the Labrador Inuit land claim. At the time of the workshop Mr. Wolfrey was a Fishery Guardian with the Nunatsiavut Government. He was a *Co‐Author* and provided input into workshop agenda and thematic areas. Active participant in all aspects of the workshop discussions, involved in writing and reviewing paper
*Leonard Auger: Bigstone Cree First Nation, Alberta Knowledge Holder* and community leader. He has served as President of the Grande Prairie Friendship Centre and co‐chaired the Indigenous Engagement Committee at the Grande Prairie Regional Hospital, Alberta Canada. He was a *Co‐Author* and provided input into workshop agenda and thematic areas. Active participant in all aspects of the workshop discussions, involved in writing and reviewing paper
*Myrle Ballard, PhD: Anishinaabe, Lake St. Martin First Natio*n, Manitoba, Canada. Associate Professor in the Department of Earth, Energy and Environment at the University of Calgary, Canada Research Chair Tier II in Indigenous Science and Sustainability Science. At the time of the workshop, she was the inaugural Director of the Indigenous Science Division (ISD) of Environment and Climate Change Canada (ECCC). She was a *Meeting Facilitator and Co‐Author*: She led the initial conceptualization and resourcing of the weather workshop. Provided input into the workshop agenda and thematic areas. She was also a workshop facilitator who participated in writing and reviewing the current paper but with no role in workshop discussions of thematic topics beyond meeting facilitation
*Keith Chaulk, PhD: Labrador Inuit* and beneficiary of the Labrador Inuit land claim. He was a Senior Navigator with ISD and he helped co‐organize and co‐facilitate the workshop and is a *Co‐Author*. He participated in writing and reviewing the current paper and served the role of *corresponding author*. He had no role in workshop discussions of thematic topics beyond meeting facilitation

The use of experts to discuss topics and collect knowledge has been supported by other researchers (Rudd et al. 2011; Martin et al. 2012; Stern and Humphries 2022). For example, Fitzsimmons et al. (2024) discuss the importance of involving Indigenous Knowledge Holders as expert researchers. It highlights the use of culturally safe methods, such as “dadirri” and “yarning circles,” to facilitate data collection and ensure that Indigenous Knowledge systems are respected and integrated into research processes. Likewise, Ciocco et al. (2024) discuss incorporating Indigenous Knowledge into climate adaptation planning and highlight the importance of engaging Indigenous Knowledge Holders through interviews and other social science methods to ensure ethical and effective collaboration.

### 2.2 Session Format

The October workshop began with a traditional Indigenous ceremony led by Solomon Wawatie, a well‐respected Indigenous Elder who participated for the duration of the workshop, grounding the discussions in cultural significance and respect for Indigenous traditions. The workshop entailed six sessions, facilitated by Indigenous staff of ISD. Each session began by introducing a weather‐related theme and opening the floor to the Indigenous Knowledge Holders to comment and discuss.

The workshop sessions were crafted to encourage open dialog and exchange of ideas. Potential agenda topics were vetted and reviewed with the Knowledge Holders prior to the workshop, and the following topics were discussed: (1) Importance of weather to traditional culture and lifestyle; (2) Traditional weather prediction methods and signs; (3) Indigenous perspectives on weather variation by season and region; (4) Indigenous perspectives on the relationship between weather and climate; (5) Weather patterns on land and their implications; (6) Weather patterns on water (marine and freshwater); (7) Indigenous perspectives on weather and its relationship with harvesting activities (hunting, fishing, berry picking, medicinal plants, etc.); and (8) Impacts of severe weather on modern Indigenous culture, lifestyle, and community.

Each session was dedicated to one of these weather topics and lasted approximately 2 h. As indicated, these topics were discussed and agreed to by the Knowledge Holders prior to the meeting and were used as a discussion guide; nonetheless, Knowledge Holders had unlimited latitude to diverge from the session topics. In terms of physical format, the Knowledge Holders consented to sit around a crescent arrangement of tables and took turns speaking. Each Knowledge Holder consented to sit in front of a microphone, and consented to session recording, which served to capture the depth and breadth of the knowledge that was shared. The role of facilitators was minimal during the sessions, limited to opening each session and seeking input from Knowledge Holders on the given session topic, as well as asking permission to end sessions in order for planned breaks such as lunch, etc. Additional time was provided on the agenda for Knowledge Holders to continue speaking on topics of their choosing. All Knowledge Holders participated in all sessions described in this paper.

Audio and visual recording services were provided by LMB Systems (Ottawa, Ontario). At the completion of the workshop, the audio recordings were transcribed by Transcript Heroes Transcription Services (Toronto, Ontario), and all files have been subsequently securely stored on the ECCC‐protected network. All third‐party contractors agreed to delete all relevant media files upon completion of each service contract. The transcribed files were subsequently provided to the Knowledge Holders and were also reviewed by the ISD co‐authors. In analyzing the transcripts, priority was given to seeking information on weather prediction, and the majority of this information was found in the session on weather prediction. Likewise, the majority of information regarding severe weather and climate was located in the session dedicated to those topics.

### 2.3 Positionality Statement

The workshop was co‐developed by the Knowledge Holders and Indigenous facilitators of ISD. Workshop logistics were primarily implemented by Indigenous staff of ISD, with occasional support from non‐Indigenous staff. Each workshop participant (Knowledge Holders, Observer, and Facilitator) consented to the use of third‐party contractors for recording and transcription services and long‐term storage of the information files and the use of their Indigenous Knowledge to produce this paper, consistent with Free, Prior, and Informed Consent. Consent letters committed to the data principles of ownership, control, access, and possession (Mecredy et al. 2018; Carroll et al. 2020) were signed by all participants. The workshop emphasized respect, autonomy, and mutual understanding. All co‐authors of this paper are Indigenous Canadians (Table 1) who participated in the workshop as either Knowledge Holders or meeting facilitators. These roles include co‐development of the agenda, as well as active roles in the writing, review, and interpretation of the results. This is consistent with autoethnographic methods used by others and reported elsewhere (Houston 2007; Mao et al. 2024). A total of eight Indigenous contributors—representing individual Anishinaabe, Cree, and Inuit perspectives—co‐authored this manuscript.

As described above, the workshop logistics (travel and event planning, audio‐visual services, consent forms, etc.) were coordinated by ISD staff, with several ISD and Meteorological Service of Canada staff attending as observers (Table 1). The workshop was conceptualized as part of broader efforts within ISD to advance Indigenous Science as distinct from, but complementary to, Western scientific paradigms, and recognizing the power imbalances within the area of Indigenous research (David‐Chavez and Gavin 2018). Pfeifer (2018) also discussed some of these imbalances in what he described as the “credibility gap.” The workshop and this paper both seek to challenge and disrupt knowledge hierarchies by supporting the co‐production of knowledge that is Indigenous‐led. Our approach centered on the voices of the Indigenous co‐authors, whose perspectives, teachings, and critiques were prioritized throughout the process. The non‐Indigenous observers and staff supported the process through coordination and documentation but did not lead thematic discussions or interpretive framing.

### 2.4 Species Names

During the workshop, Knowledge Holders shared information regarding different animal and plant species. Although a mixture of Indigenous and English language names was used, English was more commonly used during the workshop as information was being shared across cultural groups and English was the common language platform shared by all participants. To the extent possible, we have summarized species names discussed in the context of weather prediction in Table 2. To help address standardized spelling, we referenced readily available online Indigenous language dictionaries, each representing the languages of Indigenous Knowledge Holders who participated in the workshop (Table 2). In some cases, we could not find a species name in each Indigenous language. For example, we could not find a recorded name of Buffalo (i.e., American bison) in the Labrador dialect of Inuttitut. In cases such as this, it often reflects the lack of overlap between the traditional territory of a given Indigenous group and the species’ historical geographical distribution.

**TABLE 2:** Species names mentioned in the text.

Local English	Anishinaabe	Inuttitut	Cree	Binomial nomenclature
Black‐capped Chickadee	Gijigijigaaneshiinh	Atsatâtâjuk	picikîskisîs	*Poecile atricapillus*
Common Loon	Maang	Tollik	mâkwa	*Gavia immer*
Spruce Grouse	Mashkodese	AKiggilik	pihew	*Falcipennis canadensis*
Willow Ptarmigan	Pinay	AKiggivik	wapîhew	*Lagopus lagopus*
American White Pelican	Azhede		cahcakiw	*Pelecanus erythrorhynchos*
Buffalo/Bison	Mashkode‐bizhiki		paskwâwimostos	*Bison bison*
Ringed Seal		Natsik	âhkik	*Pusa hispida*
Moose	Mooz	Tuttuvak	môswa	*Alces alces*
Elk	omashkooz		wâwâskesiw	*Cervus canadensis*
Dogberry tree	Makominagaawanzh		waciwahtik	*Sorbus americana, Marshall*
Red Berry		kimminak		*Vaccinium vitis‐idaea* , L

*Note:* Relevant online language resources are listed below the table. Shaded cells indicate that the species' name was absent from the online resources. Anishinaabe: the Ojibwe People's Dictionary (Treuer et al., n.d.). Inuttitut: the Labrador Virtual Museum (Them Days Inc., n.d.). Cree: the Online Cree Dictionary (Miyo Wahkohtowin Community Education Authority and Waugh, n.d.). Note in the case of Cree dialects, we typically use the form and spelling credited to the Alberta Elders Cree dictionary (source: LeClair et al. 1998); this dialect is referred to as nêhiyawêwin (i.e., Plains Cree). Scientific binomial: the Integrated Taxonomic Information System (Integrated Taxonomic Information System, n.d.).

## 3 Results

### 3.1 Climate Change and Severe Weather

Analysis of the transcript indicates climate change was mentioned more than 100 times, making it the most discussed theme during the workshop. While this was in part due to a dedicated session on climate change, the climate theme also emerged across all other sessions regardless of focus. Table 3 summarizes the sub‐themes within the discussions of climate change, and these include: *Impacts on Traditional Practices*, such as disrupted seal harvesting due to altered ice and snow conditions and changing bird migration patterns that affect hunting and gathering. *Ecosystem Shifts* were observed and discussed, including the northward movement of southern species like pelicans, snakes, and salamanders, as well as declines in moose, deer, fish, and bear populations due to a combination of habitat destruction and climate stress. In the context of climate, *Waterway Concerns* were linked to variability in water levels, reduced fish availability, and also contamination by industrial pollutants like methyl mercury. Participants also discussed *Unpredictable Weather Patterns*, such as intense storms and shorter cold seasons that interfere with ice road access and traditional activities. *Challenges to Adaptation* include economic stress and unsafe travel conditions, especially in remote communities. Climate change was further tied to impacts on *Health and Well‐Being*, where reduced access to land‐based practices and traditional foods contributed to emotional and physical health concerns. Finally, participants emphasized the *Cultural and Social Impacts of a changing climate*, noting that disrupted relationships with the land threaten cultural continuity and community cohesion. More detailed examples and context for these observations are provided through Knowledge Holder quotes in the relevant portions of the discussion.

**TABLE 3:** Summary of concerns regarding impacts of climate change.

Category	Key findings
Impacts on Traditional Practices	Altered ice and snow conditions disrupt timing and extent of seal harvesting Changes in bird migration patterns affect hunting and gathering Increased need for seasonal adaptability due to fluctuating weather and animal behavior
Ecosystem Shifts	Southern species (e.g., pelicans, snakes, salamanders) are appearing in colder regions, reflecting climate shifts Wildlife reductions (e.g., fish, moose, deer, elk, bears) caused by habitat destruction, pollution, and climate‐related changes
Waterway Concerns	Declining and variable water levels impact water quality and fish availability Industrial contamination (e.g., methyl mercury) harms aquatic ecosystems and species like seals
Unpredictable Weather Patterns	Shorter cold spells, intense storms, and changing ice road seasons disrupt traditional activities and remote supply chains
Challenges to Adaptation	Weather variability affects safety during travel and participation in traditional practices Economic pressures and climate unpredictability hinder adaptation in remote communities
Health and Well‐Being	Reduced land‐based activities disrupt emotional, mental, physical and spiritual health Lack of access to wild foods increases health risks in isolated communities
Cultural and Social Impacts	Disruption of Indigenous people's relationship with the land negatively impacts cultural practices and social cohesion

Severe weather was another major theme that received significant attention throughout the workshop (Table 4). While it had a dedicated session, the impacts of severe weather were also raised in other sessions. Several important sub‐themes emerged. *Emergency Response Disparities* were a central concern, with participants highlighting that Indigenous communities face significant gaps in emergency preparedness and response compared to non‐Indigenous communities. These gaps include insufficient firefighting equipment, limited access to clean water, and inadequate emergency infrastructure, all of which heighten risk during extreme weather events. *Impact on Traditional Activities* was also raised in the context of severe weather. Participants noted that severe weather events increasingly disrupt traditional land‐based activities such as hunting, fishing, and berry picking, leading to growing food insecurity. There was also concern that the loss of reliable seasonal cues undermines existing Knowledge, threatening both cultural continuity and community resilience.

In addition, the Knowledge Holders discussed topics like the epistemology of Indigenous Science and Traditional Knowledge. They shared their thoughts on the need for collaboration across knowledge systems, along with the importance of Indigenous Knowledge and Indigenous Science in policy development and decision‐making, especially where they impact Indigenous communities.

**TABLE 4:** Summary of concerns regarding severe weather.

Severe weather	
Emergency Response Disparities	Indigenous communities experience significant disparities in emergency response compared to non‐Indigenous communities Lack of resources like firefighting equipment, water systems, and emergency management infrastructure increases risks during severe weather
Impact on Traditional Activities	Severe weather disrupts traditional practices such as hunting, fishing, and berry picking, leading to food insecurity Loss of Traditional Knowledge about weather patterns threatens cultural preservation and community resilience
Environmental Changes and Industrial Impacts	Hot and dry conditions prolong fire seasons, with some regions experiencing late‐season wildfires Record‐breaking floods displace thousands, damage homes, and disrupt communities Industrial activities exacerbate vulnerabilities to extreme weather
Need for Resources and Policy	Indigenous communities require improved emergency management systems and infrastructure Legislative reforms and stronger policies are needed to address environmental degradation and cumulative impacts from industries like agriculture, forestry, and mining

The sub‐theme of *Environmental Changes and Industrial Impacts* included references to prolonged fire seasons driven by hotter and drier conditions, late‐season wildfires, and record‐breaking floods that have displaced residents and destroyed homes. Industrial activity, particularly in agriculture, forestry, and mining, was seen as compounding the risks and consequences of these extreme weather events. Finally, *the Need for Resources and Policy* was widely emphasized with Knowledge Holders calling for stronger emergency management systems tailored to Indigenous needs, as well as legislative reforms and environmental policies that better address the cumulative effects of climate and industrial pressures on Indigenous lands and communities. More detailed examples and context for these observations are provided through Knowledge Holder quotes in the relevant portions of the discussion.

### 3.2 Weather Predictions

**FIGURE 2:**
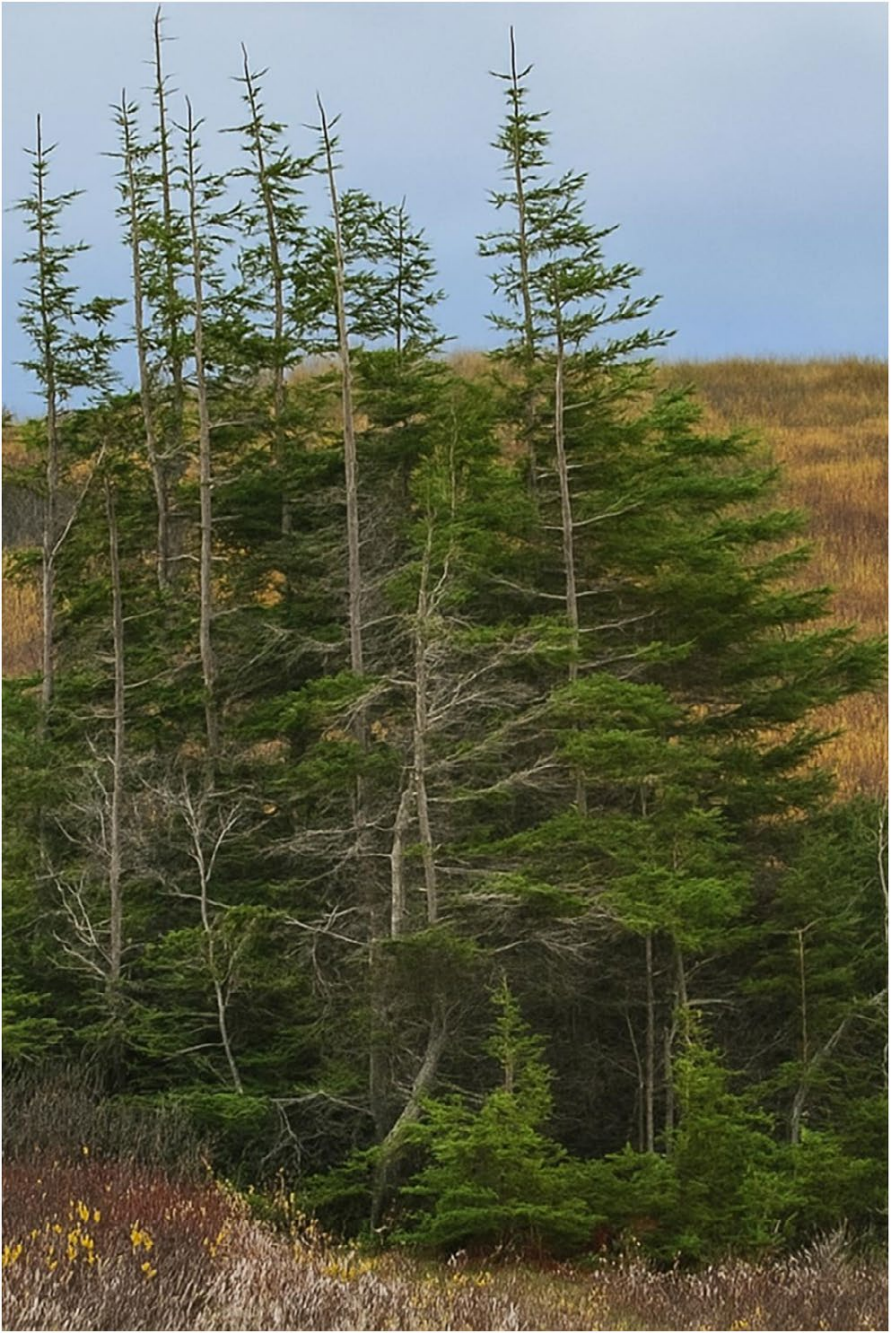
Photograph of a small group of black spruce trees ( *Picea mariana* , Mill) located in Churchill, Manitoba, Canada (approximately 58.76 N, 94.16 W). The image shows trees with bare trunks on the northerly side and larger amounts of branches and needles on the southern exposure. Photo provided by Dr. Myrle Ballard (October 2024).

Table 5 summarizes various weather prediction signs discussed by the Knowledge Holders. These include examples of observed animal behavior, plant growth, natural phenomena, and other indicators. Key observations include animal behaviors used to predict changes in weather. In some cases, patterns of plant growth in relation to long‐term environmental influences were discussed (Figure 2). In addition, signs related to natural phenomena—such as sundogs (i.e., parhelia) and atmospheric patterns during sunrise and sunset—are discussed in the context of weather prediction (Table 5). For example, weather signs related to atmospheric refraction, barometric pressure, and patterns of plant growth were provided and discussed in detail.

**TABLE 5:** Summary of bridging and braiding of weather prediction signs, Ottawa, October 2023.

Item no.	Category	Type	Bridging: What we heard	Braiding: Pathway(s)
1	Animal	Chickadee	Presence or absence in the morning can be used to indicate weather	Barometric and/or temperature‐related
2	Animal	Loon	Behavior and vocalizations are indicators of wind and bad weather	Barometric: sensitivity to atmospheric pressure changes may impact behavior
3	Animal	Spruce Grouse and Willow ptarmigan Crop	The crop (a digestive organ) of a spruce grouse or willow ptarmigan, if full at time of death, indicates bad weather	Energy requirements in relation to temperature and environmental conditions
4	Animal	Spruce Grouse Breastbone	Boiled Breastbones of spruce grouse will darken if bad weather is approaching	Barometric: Gas exchange across thin tissues
5	Animal	Buffalo	Thick hide and hair suggest a cold winter	Physiological responses to temperature
6	Plant	Dog Berry Tree Berries	Abundance of berries on the Dogberry tree (American Mountain Ash) can be used as a predictor of snowfall for the upcoming winter season	Temperature/Precipitation: energy requirements in relation to temperature and environmental conditions in preceding seasons
7	Plant	Plant Orientation	On tundra and other landscapes, plants will often grow in certain directions	Reflects local environmental conditions and long‐term climate trends, which affect patterns of plant growth
8	Plant	Red Berry	These plants are only suitable to be picked after the first frost. The frost forces an insect larva to emerge from the plant	Temperature‐induced plant and insect phenology
9	Natural	Joint Pain	Potential predictor of weather changes	Barometric: gas exchange across bone, cartilage and/or body fluids trigger sensory nerves associated with pain
10	Natural	Looms (Mirage)	Distant objects appear closer, indicating wind or bad weather coming. Like Fata Morgana, a form of mirage that occurs in deserts, polar regions, or over large bodies of water	Atmospheric Refraction: temperature inversions cause atmospheric lensing. Alternating layers of cold and warm air can lead to windy conditions as air molecules move across temperature layers
11	Natural	Sundogs (Parhelia)	Predictors of weather changes	Atmospheric Refraction: Ice crystals in the atmosphere refract light
12	Natural	Sunrise/Sunset	Predict upcoming weather during sunrise and sunset based on colors and patterns in the sky	Atmospheric Refraction: Raleigh scattering and other cues
13	Other	Boat Wake	Calm water in boat wakes during windy conditions indicates wind will die out. If waves persist in the boat wake, it means the wind will continue to blow	Wind/Tide/Cavitation: unclear mechanism, could be related to energy thresholds needed for wave forms to propagate or re‐emerge once the wave form is broken by boat wake
14	Other	Candle	Long and thin flame for clear weather, short and fat flame for rain or snow, and flame jumping around for wind	Barometric: flame shape changes in response to atmospheric pressure
15	Other	Woodstove	The shape and behavior of smoke column exiting the chimney as an indicator of weather, especially when wind is calm	Barometric/Wind: smoke column changes shape in response to atmospheric pressure and/or wind

## 4 Discussion

Two important goals of this project were to elevate the voices of Indigenous Peoples and to increase awareness of Indigenous Science, with both of these goals being important components of *Bridging*. Based on post‐event feedback, the workshop was well received by the Knowledge Holders and observers, who welcomed the opportunity to gain experience and share their knowledge with one another. While the pool of Knowledge Holders was small, it was diverse, with individuals from multiple Indigenous cultures present, representing different regions of Canada, and including both female and male voices. A common comment was that the smaller workshop enabled each Knowledge Holder more time to share their expertise, which supported deeper dives into any given topic.

### 4.1 Cultural Context

Many scholars have argued that the socio‐cultural milieu in which research occurs can affect its application and interpretation (Nisbett et al. 2001). For example, Graham (1987) explored how Soviet ideology shaped biological science, particularly the rejection of Mendelian genetics in favor of theories that emphasized collective transformation, thus aligning with Marxist ideas of social transformation. Likewise, Lewontin and Levins (1985) argued that capitalism affects Western interpretation of evolution by focusing on competition. Meanwhile, Latour and Woolgar (1986) showed how the interpretation of empirical data in laboratories is shaped by cultural norms, language, and negotiation among scientists, and Nisbett et al. (2001) argued that East Asian cultures tend to interpret evidence holistically, while Western cultures use more analytic methods, influencing how conclusions are drawn from the same data. The goal of this paper is not to solve these epistemological and philosophical discussions but rather to help demonstrate Indigenous Science in practice through the topic of weather. Nonetheless, many topics that were discussed by the Knowledge Holders revealed a holistic world view and sometimes fell into the realms of epistemology, metaphysics, governance, politics, policy, and planning.

For example, many of the Knowledge Holders highlighted that Indigenous weather science is holistic and discussed that weather patterns are highly interconnected to the natural environment. They discussed the cultural significance of weather and how it shapes daily life, including the profound influence it has on Indigenous cultures and traditions. They also stressed the importance of maintaining a balance between spirituality and environmental awareness. Further to this, the general view of the Knowledge Holders was that weather is not an isolated system, but rather, it interacts with biophysical landscape, human culture, and human health.

Like I said, our culture is holistic. Can't really take it apart. And when you start talking about weather you have to talk about the other parts as well, including our spirituality, our ecosystem, and our social, cultural economics. (Indigenous Knowledge Holder, Ottawa, October 2023)

We also heard how Indigenous Knowledge Holders are recognized as experts by community members with similar expertise and experience. This community‐based endorsement of expertise is often grounded in known examples of performance and repeatability demonstrated in real‐world activities. While rarely framed as such, it can be easily argued that Indigenous Knowledge Holders, through this third‐party endorsement, undergo peer review.

These are things stuff that I learn. I might not have a degree. But my knowledge determines my degree. (Indigenous Knowledge Holder, Ottawa, October 2023)

### 4.2 Environmental Change

Workshop discussions heavily featured climate change, particularly its impact on traditional practices and animal behavior. For example, ice and snow conditions can impact the timing and extent of ringed seal whelping (Table 5), which can impact the timing and extent of seal harvesting. Likewise, there was discussion related to the timing of migration of various bird species. Knowledge Holders noted significant shifts in local ecosystems, including the appearance of southern species like pelicans, snakes, and salamanders in traditionally colder regions, indicative of shifting climates. A common concern was the reduction in wildlife in some regions of Canada with respect to various species of fish, but also moose, deer, elk, and bears. These changes were attributed to various factors, including habitat destruction and pollution from industrial activities such as pulp mills and oil fields. Likewise, water levels in lakes and rivers have been observed to decrease and/or become highly variable, impacting the quality of water and the availability of fish, a staple in many Indigenous communities' diets. Concerns were also heard about the contamination of waterways by industrial activities further exacerbating these impacts, affecting both human and wildlife populations.

So, with that there's methyl mercury coming out because of the trees that are in the flooded areas which somebody mentioned bioaccumulation – I think was you – this morning. That's happening to us there because the small microorganisms is – the fish the small fish are eating the microorganisms coming up. The top one next to us are the seals. Seals. One of the most important animals to us probably impacted most by the Hydro developments because of the methyl mercury coming out. (Indigenous Knowledge Holder, Ottawa, October 2023)

One important message seemed to be that a changing climate has affected once well‐established patterns in weather, which means animal behavior and the timing of Indigenous harvesting practices are now fluctuating more widely from year to year, requiring higher levels of seasonal adaptability. Similar observations have been reported by others (Turner and Clifton 2009; Weatherhead et al. 2010; Downing and Cuerrier 2011; Simonee et al. 2021; Bishop et al. 2025). The Knowledge Holders reported that the unpredictability of weather patterns, including shorter cold spells in winter and more intense, shorter‐duration storms, has made traditional activities like hunting, fishing, and gathering increasingly difficult. The change in ice road seasons, essential for transportation and supply chains in remote northern communities, underscores the tangible impacts of climate change on daily life.

I think that's a good point that you make that we don't know if they're true [i.e., the traditional signs used to predict safe travel on the land] today now because of climate change. The signs are changing. And the weather is getting more and more extreme. (Indigenous Knowledge Holder, Ottawa, October 2023)

Also discussed was the fact that owing to the nature of the modern economy (i.e., planned work leave) or owing to the predictability of ice thickness, it is not always possible for community members to adapt to highly variable weather and climate. Consequently, there are times when participation in traditional practices is being negatively impacted. In small, northerly isolated communities, this can have many impacts. For example, the extremely high price and limited availability of store‐bought food often necessitate the harvesting of wild food for subsistence and/or survival purposes. The inability to harvest due to unpredictable weather events and climate change can lead to both direct and indirect health problems (Cajete 2000). It was reported that the inability to do land‐based practices can negatively impact emotional and mental well‐being, or, said another way, people were often happiest when out on the land (Cunsolo and Ellis 2018). Similar issues and concepts have been raised in other research (e.g., Fuentes et al. 2020; Ford et al. 2020).

Another impact of severe weather regarding people's health the culture is impacted everything is impacted. The social fabric of the community is totally disrupted. And there's a lot of deaths too from being displaced from the land because we all say like we know that Indigenous people are so close to the land when you take that close relationship away from the land it's going to impact you mentally emotionally physically. (Indigenous Knowledge Holder, Ottawa, October 2023)

### 4.3 Severe Weather

The Knowledge Holders also discussed the consequences of severe weather events on modern Indigenous communities, emphasizing the need for effective emergency response measures. They also highlighted disparities in emergency response between Indigenous and non‐Indigenous communities in Canada. The discussions underscored a critical need for resources and infrastructure to cope with severe weather events. The lack of adequate firefighting resources, water supply systems, and emergency management infrastructure in many Indigenous communities amplifies the risks associated with extreme weather. Furthermore, severe weather events disrupt traditional activities such as hunting, fishing, and berry picking, impacting cultural practices and food security. The loss of Traditional Knowledge about weather patterns and environmental changes was also a concern, highlighting the need for cultural preservation amidst changing climates.

Severe hot weather actually dried up the forests and it's been getting worse every year… we were still under high fire conditions in Grande Prairie at the start of October and that is really unusual for our area. (Indigenous Knowledge Holder, Ottawa, October 2023)

I see the impacts of industry and development…it's disconcerting because last year we were flooded. The worst flood in our history; over 2000 people displaced. Hundreds of homes ruined and lots of – and it's all because of agriculture. And I look all around us, the cumulative effects that impact our nation. Forestry. Aggregate. Mining. You name it. The impacts are there; legislation and policy. (Indigenous Knowledge Holder, Ottawa, October 2023)

### 4.4 Bridging and Collaboration

The ISD (2024) describes *Bridging* as processes and activities that bring together Indigenous and Western Science to convey mutual awareness and respect. In this regard the workshop was an engagement activity that served to bring together Indigenous and Western scientific views and to convey mutual awareness, which aided relationship building between the Knowledge Holders and the workshop observers. Furthermore, workshop reports and subsequent knowledge products such as this paper are learning resources that, ideally, will support the development of secondary learning tools or future *Braiding* and *Weaving* projects (see examples in the section below).

Overall, the Knowledge Holders emphasized the importance of collaboration and mutual respect between Indigenous and western scientific knowledge systems, which is the essence of Bridging within the framework of *Bridging*, *Braiding*, and *Weaving*. Knowledge Holders also discussed the importance of both preserving and *Weaving* Indigenous Science with Western science and technology to address challenges posed by climate change.

We're bringing the two knowledges together: the Indigenous scientist and the western scientists… Because a lot of the knowledge that Indigenous people, the Indigenous scientists have, the Knowledge Holders, our knowledge of the patterns, seasonal patterns, birds, the way birds behave, the way animals behave… And that these are the kind of knowledges we want to bring together so that we can learn from each other and develop new policies, work together to best manage the land and the environment. (Indigenous Knowledge Holder, Ottawa, October 2023)

Nonetheless, it remains a challenge to understand the extent that collaboration between Indigenous Knowledge Holders and Western‐trained scientists can actually occur. For those trained within a Western paradigm, Indigenous worldviews may seem distinct and markedly different (and vice versa). However, for projects that impact Indigenous Peoples, this tension underscores the importance of *Bridging*, *Braiding*, and *Weaving*, each of which provides opportunities for Indigenous Knowledge Holders to be included in research processes ranging from conceptualization to implementation, interpretation, and dissemination (Bartlett et al. 2012; Johnson et al. 2016; Pfeifer 2018). This is especially true, given that the majority of global research funding is awarded to Western‐trained researchers. In such cases, those leading research projects that intersect with Indigenous rights and interests have a responsibility to champion diverse, inclusive teams that authentically involve Indigenous experts. Such inclusion should help to dismantle systemic barriers that limit the participation of Indigenous Knowledge Holders and foster culturally responsive and responsible research practices, including the interpretation of results as well as the development and application of evidence‐based policy (Smith 2012; Tobias et al. 2013). Furthermore, meaningful participation by Indigenous community members in all phases of research enhances trust, builds relationships, and increases the likelihood of research findings being accepted and applied locally (Koster et al. 2012).

It should be noted that while Indigenous Peoples hold a deep appreciation for the value of Indigenous Science and Indigenous Knowledge (Cajete 2000; Snively and Corsiglia 2000; Johnson and Murton 2007) and can also display a high degree of adaptability integrating Indigenous and Western scientific approaches (Mugambiwa 2018), a potential benefit of collaboration is the facilitation of two‐way knowledge transfer. This begins with *Bridging*, wherein Western scientists and Indigenous Peoples develop mutual awareness and respect for each other's ways of knowing. Over time, *Bridging* may evolve into *Braiding*, where insights from both knowledge systems are not only exchanged but also co‐produce new theoretical understandings, a process that can inform both Western and Indigenous Science frameworks (Reid et al. 2021; Whyte 2013) and eventually leading to fully woven projects.

However, even in ideal settings, challenges persist, particularly regarding power imbalances between Indigenous and Western scientific paradigms. Globally, Western science retains significant influence, and when these systems are brought together, there is a risk that the dominant framework may subsume or marginalize Indigenous ways of knowing. In other cases, frameworks designed to promote collaboration and synergy may be misused by bad‐faith actors, leading to extractive rather than equitable outcomes (Simpson 2004; Smith 2012). In addition, Indigenous Knowledge Holders still encounter various roadblocks from western gatekeepers and policymakers (Snively and Corsiglia 2000). Fortunately, there is a growing number of Indigenous scholars working to highlight Indigenous Science, presenting compelling cases for its utility and relevance (Ballard 2012; Kimmerer 2021; Chaulk et al. 2013; Pfeifer 2018; Hill 2020; Neeganagwedgin 2022). Further efforts are needed to increase the visibility, awareness, and respect for Indigenous Science as there is still much work to be done, as decision‐makers struggle with new and emerging requirements to incorporate Indigenous Science and Indigenous Knowledge into their western‐based practices (Nadasdy 1999; Parter and Skinner 2020).

### 4.5 Braiding

The ISD of ECCC describes *Braiding* as the intertwining of Indigenous and Western science for a more comprehensive understanding (ISD 2024). As an example, all Indigenous Knowledge Holders reported using modern weather reports in combination with their own weather prediction methods. This reflects the growing movement of Two‐Eyed Seeing (Bartlett et al. 2012) that braids elements of Indigenous and Western Science. This was especially true both for those preparing to travel and while actively traveling out of the land. This intertwining of information sources demonstrates the adaptability of the Knowledge Holders and encapsulates *Braiding* in daily life.

While all workshop sessions provided important information, the session on weather prediction methods yielded some opportunities to highlight examples of *Bridging*, *Braiding*, and *Weaving*. This session discussed various signs used by Indigenous Knowledge Holders to assist in weather prediction, including observations of animal behavior, interpretation of patterns in plant and tree growth, and atmospheric conditions. The most prominent examples are summarized in Table 5, and of these we select several examples where further *Braiding* and *Weaving* could be explored.

#### 4.5.1 Barometric Pressure

During the workshop, we heard examples of weather signs that seem to be related to barometric pressure (Table 5). For example, the behavior of some bird species (i.e., loons and chickadees) prior to the onset of a weather system, joint pain in individuals, and the behavior of indoor candle flames. Each of these weather signs, in whole or in part, is likely related to barometric pressure changes, though follow‐up work would be required to co‐develop projects to explore these weather signs more fully.

In addition to these examples, an intriguing method of weather prediction was described, involving the breastbone of a cooked spruce grouse (Table 5, Item 4). The breastbone is sometimes referred to as the keel or boat. After boiling and consuming the meat, changes in the color of the keel are sometimes observed. Specifically, if the keel's color darkens and becomes more mottled, it signals the onset of bad weather.

In terms of *Braiding* these observations with Western science, it is important to know that bird bones are often thin and hollow, features thought to have evolved to reduce body weight and enable flight (Dumont 2010; Sullivan et al. 2017). We suggest that these reported color changes after cooking result from gas exchange across thin and/or hollow bone materials (Guedj and Weinberger 1990; McAlindon et al. 2007; Bennett et al. 2012). In essence, the keel complex acts as a barometer. However, it is unclear what role cooking the breastbone plays in mediating this color change process, including how it affects the gas exchange pathways. However, it was reported that this process only works with a freshly harvested bird, as compared to birds that have been killed, frozen, and then cooked.

#### 4.5.2 Atmospheric Refraction

During the workshop, we also heard of several examples of weather prediction signs that are likely related to atmospheric refraction, including looms and sundogs (Table 5). Looms are said to occur when distant objects appear closer to the observer than they actually are. Looms are similar to Fata Morgana, a complex type of superior mirage that occurs in deserts, polar regions, or over large bodies of water, usually caused by strong atmospheric temperature inversions and which can be associated with the onset of additional weather features related to wind and calm conditions (Andrews 2010; Young 2015). Meanwhile, sundogs are bright spots that appear on either side of the Sun, typically when it is low on the horizon. They form when sunlight is refracted through hexagonal ice crystals in high‐altitude cirrus or cirrostratus clouds, producing colorful patches, often red on the side closest to the Sun and blue further out (Minnaert 1993; Lynch and Livingston 2001). Sundogs are sometimes seen ahead of an approaching low‐pressure system, especially in winter, as cirrostratus clouds can indicate an incoming warm front or storm (Ahrens and Henson 2019).

One Indigenous Knowledge Holder described a method of weather prediction involving the sunrise/sunset, which they had learned from an Elder many years ago. The Knowledge Holder began by citing the “red sky at night” rhyme as a weather mnemonic (where a red sky at night means good weather and a red sky in the morning means bad weather) (Goliger and Milford 1998; Lynch 2008).

But the Knowledge Holder went on to further explain that there is even more info that can be gained by watching sunrise and sunset events in real time. That information is gained by observing the various colors and diffusion patterns that occur during the actual moments of sunrise and sunset beyond the simple red sky mnemonic. With practice and training, these patterns can then be used as indicators of impending weather. From a *Braiding* perspective, the visual cues discussed by Knowledge Holders are likely based on Rayleigh scattering, which is the name of a naturally occurring process where wavelengths of light interact with particles in the atmosphere (Fröhlich and Shaw 1980). The size of the atmospheric particles relative to the wavelength of light will produce different colors (Bucholtz 1995). Rayleigh scattering is currently used in many Western scientific fields, including physics, astronomy, and engineering, to measure the temperature, velocity, and density of air particles.

However, we suggest that the important insight here is related to the timing of the observation. Sunset and sunrise both represent unique times of the day, as both are periods of phase transition (Verhulst and Stankov 2017). Phase transitions have been recognized as important in a number of different physical sciences (Shrager et al. 1987; Papon et al. 2002; Alberti 2017), including atmospheric science (Verhulst and Stankov 2017). The authors suggest that during the transition period from daylight to night and vice versa, novel weather information (beyond the simple red sky) is revealed through Rayleigh scattering. This information is available to a trained observer and potentially could even be used by remote sensing platforms for use in weather prediction. Additional research is required from both Western and Indigenous Science perspectives to more fully understand the practicality, applicability, and utility of weather predictions based on visual observations at sunrise and sunset, including opportunities for machine learning.

### 4.6 Weaving

The ISD of ECCC describes *Weaving* as the application of Indigenous methodologies and paradigms alongside Western approaches to research and with application to policy development and decision‐making (ISD 2024). Within the *Bridging*, *Braiding*, and *Weaving* framework, *Weaving* is the most complex. It can be difficult for a small group to have the expertise to fully weave a project, and, as such, it will often require a multi‐ and interdisciplinary approach to achieve. With this said, in this section we suggest a woven project based on information shared during the workshop.

#### 4.6.1 Plant Growth and Climate Patterns

Several Knowledge Holders explained (i.e., *Bridging*) that in some regions of northern Canada, some plant communities can exhibit specific patterns of growth. For example, the branches, needles, leaves, and even trunks of plants can orient toward the south (see Figure 2), including small trees, ground plants, and lichens. These patterns of plant growth have been used by Indigenous people for millennia as a method of wayfinding in northern regions. To understand this dynamic (i.e., *Braiding*), the process of thigmomorphogenesis has been well documented throughout the plant kingdom (Jaffe 1973; Chehab et al. 2009), and it is defined as occurring when growth patterns change in response to mechanical factors such as wind, snow, etc. In the case of subarctic black spruce trees ( *Picea mariana* ), sometimes referred to as *Krummholz* forest (Holtmeier 1981; Albertsen et al. 2014), trees shift from apical/upward growth to lateral and/or prostrate growth; in some cases, these can be referred to as flag trees. In subarctic regions of Canada, *Krummholz* can often have a pronounced directionality owing to the direction of prevailing winds (Albertsen et al. 2014).

To understand this observation more fully, it is important to consider how plants grow. Environmental factors play a crucial role in influencing plants' energy needs and growth strategies (Jonasson et al. 1999). Previous studies have shown that plants adjust their growth patterns to optimize sunlight exposure while minimizing the impact of climatic stressors (Chapin 1991). It is possible that the large‐scale orientation of plant communities is occurring in part as a response to long‐term climate trends. We theorize that these growth patterns may shift as the typical conditions of northern environments change. For instance, Bokhorst et al. (2010) observed that subarctic plants could begin spring growth prematurely during brief winter warm spells.

We propose integrating Indigenous Science with Western science to establish a comprehensive and fully woven research program. This program aims to assess the ecological impacts of climate change by documenting baseline plant growth patterns, including any changes in the orientation of northern plant communities over time. If the pattern is simply a reflection of latitude and angle relative to the sun, the pattern should not change over time. However, if these plant growth patterns are also partly in response to stresses from a cold climate, then changes could occur with shifts in climatic conditions. For example, during the Eocene, the flora and fauna of the Canadian High Arctic were quite different from today (Eberle and Greenwood 2012).

To outline a potential woven project, the co‐authors propose beginning with interviews of Indigenous Knowledge Holders in northern Canada. These discussions would aim to pinpoint locations where the orientation of plant communities is most noticeable (i.e., tree line). Following this, a variety of techniques would be employed to record data on plant growth, including aspects such as orientation, biomass, and community composition. In collaboration with local Indigenous communities, a comprehensive, long‐term program would be set up to monitor these naturally occurring patterns. This would involve Indigenous team members collaborating with Western scientists, including botanists and climate experts, to gather, analyze, and report the data. The potential benefits are clear. Not only could this serve as a practical tool for environmental monitoring, but it also offers a valuable opportunity to explore the processes of *Bridging*, *Braiding*, and *Weaving* Indigenous and Western scientific processes and provide opportunities for training youth in both Western and Indigenous Science methods.

## 5 Conclusions

The workshop provided an initial opportunity to document and raise awareness of Indigenous Weather Science and insights with respect to environmental change from across Canada (i.e., *Bridging*). The small group setting provided each Knowledge Holder with the opportunity to discuss their weather knowledge in a safe and respectful setting. Despite the valuable insights gained through our workshop, we recognize that our findings represent only a fragment of the vast array of weather prediction methods employed by Indigenous Peoples across Canada. The diversity of these methods varies not only between different Indigenous groups but also within the regions, particularly small and remote communities that maintain strong ties to the land and are most likely to possess unique and insightful examples of Indigenous Science pertaining to weather prediction. This assertion is underscored by an enlightening piece of knowledge shared by one of the expert Knowledge Holders after our initial workshop. They recounted,

Many years ago, when my wife's Elder uncle was still with us, I walked into his place on reserve at God's Lake and made the comment in Cree that it has been a cold summer. To which the uncle replied, ‘the water is high, that is why.’ And we live mostly close to the lakeshore so we notice whether the water is high or low, compared to other years I suppose. Anyway, when the Elder made that comment about it being a cold summer and the water level being high, my first thought was, ‘what does the water level and the cold or warm weather of summer have to do with each other?’ I did not have to ask as he went on to explain, when the water is high, it covers up most of the rock surface on the reefs and shores of the lake, so the sunlight cannot bounce off the rock surfaces to heat up the atmosphere. And you can conclude that the sunlight is soaked up by the water during high water summers and so we end up with ‘cold’ summers….

It is obvious to the co‐authors that there remains much Indigenous Science information to be shared and explored with respect to understanding weather patterns. Sharing knowledge is the first and most fundamental step in the *Bridging*, *Braiding* and *Weaving* framework. As previously described, *Bridging* is a process of building connections, and it requires openness to Indigenous perspectives and evidence. Only once *Bridging* has occurred can we move onto *Braiding* and *Weaving*. *Braiding* represents the next level of complementarity, where Indigenous and Western knowledge systems are intertwined to enrich understanding. In the best cases this approach can help find synergies and shared insights that can lead to more comprehensive environmental solutions.

The final phase is *Weaving*, and it involves the full participation of Indigenous scientists and Knowledge Holders throughout the research process, from design to implementation, analysis, and reporting. It embodies a complete intersection of methodologies, grounded in respect for and application of Indigenous paradigms and tools alongside Western approaches. *Weaving* goes beyond collaboration, embedding Indigenous methodologies and worldviews into the fabric of environmental research and policymaking.

The weather workshop, along with subsequent reporting, are exercises in *Bridging*, and the workshop discussions highlight the potential of participatory approaches to enhance our understanding and response to environmental challenges. By *Bridging*, *Braiding*, and *Weaving* Indigenous and Western science, the Knowledge Holders hope to bolster the credibility of both knowledge systems and ensure that environmental stewardship is inclusive, holistic, and effective. For example, in instances where Indigenous and Western Science align, it validates the strength and reliability of mutual conclusions. Conversely, discrepancies between these knowledge systems should prompt further investigation, suggesting areas where understanding may be improved. By advocating for this complementary and cooperative approach, we aim to foster a more inclusive, effective, and respectful framework for addressing the pressing challenges of climate change and ecological degradation. The promise of this synthesis lies not only in the enhancement of scientific outcomes but also in the advancement of a path toward reconciliation.

Beyond areas for future research, it might be possible to weave various weather prediction signs in practical real‐world settings. For example, follow‐up work could be undertaken to explore the use of weather prediction signs to aid survival training for backcountry hikers, military or search and rescue workers, especially when these individuals are in situations where they are cut off from modern telecommunications. It is suggested that additional collaboration with Indigenous Knowledge Holders is warranted to better understand how to document the full array of weather prediction signs used by Indigenous people across Canada and to better understand their implications and potential uses. This type of documentation can help preserve Indigenous Science for future generations and make it more accessible in the classroom to Indigenous students.

## Author Contributions

**Keith Chaulk:** conceptualization (supporting), data curation (lead), formal analysis (equal), funding acquisition (supporting), investigation (equal), methodology (equal), project administration (supporting), resources (supporting), software (supporting), supervision (supporting), validation (equal), visualization (equal), writing – original draft (equal), writing – review and editing (equal). **Myrle Ballard:** conceptualization (lead), data curation (supporting), formal analysis (equal), funding acquisition (lead), investigation (equal), methodology (equal), project administration (lead), resources (lead), software (supporting), supervision (lead), validation (equal), visualization (equal), writing – original draft (equal), writing – review and editing (equal). **Stewart Hill:** formal analysis (equal), methodology (equal), validation (equal), visualization (equal), writing – original draft (equal), writing – review and editing (equal). **Mina Campbell:** formal analysis (equal), methodology (equal), validation (equal), visualization (equal), writing – original draft (equal), writing – review and editing (equal). **David Wolfrey:** formal analysis (equal), investigation (equal), methodology (equal), validation (equal), writing – original draft (equal), writing – review and editing (equal). **Len Auger:** formal analysis (equal), investigation (equal), methodology (equal), validation (equal), writing – original draft (equal), writing – review and editing (equal). **Solomon Wawatie:** formal analysis (equal), investigation (equal), methodology (equal), validation (equal), writing – original draft (equal), writing – review and editing (equal). **Mike Sutherland:** formal analysis (equal), investigation (equal), methodology (equal), validation (equal), writing – original draft (equal), writing – review and editing (equal).

## Conflicts of Interest

This research and the associated workshop were funded by Environment and Climate Change Canada (ECCC) through the Indigenous Science Division. The funding supported workshop organization, participant travel, venue fees, third‐party services, and collaborative writing activities. The authors declare no conflicts of interest related to the research, writing, or publication of this article. While some authors were affiliated with ECCC at the time of the workshop, all contributions reflect their personal perspectives and professional expertise and do not necessarily represent the views of ECCC. The views expressed in this article are those of the authors and do not necessarily reflect the official policies of ECCC or the Government of Canada.

## Data Availability

As described in the methods, this study is based on Indigenous knowledge and adheres to the principles of Ownership, Control, Access, and Possession (OCAP) and Indigenous data sovereignty. As such, access to the data generated and analyzed during this study is subject to these principles and is available from the corresponding author upon reasonable request, with appropriate consideration given to Indigenous governance and community protocols. This paper is grounded in knowledge shared during a weather workshop that was co‐developed with Indigenous Knowledge Holders. In keeping with the principles of Free, Prior, and Informed Consent (FPIC) and the United Nations Declaration on the Rights of Indigenous Peoples (UNDRIP), the original ownership of the knowledge remains with the Knowledge Holders and their respective communities. The insights shared were part of a relational and context‐specific process, shaped by trust, prior relationships, and the intent of collective learning. As such, the data cannot be separated from the people, place, and context in which it was shared. The Knowledge Holders have provided explicit consent to publish the information presented in this article. While the information included here is shared with permission, the broader data and discussions from the workshop are not publicly available. Any future access or use of workshop‐related materials must be negotiated in consultation with the Knowledge Holders.
